# Supplementary material for: Modeling oxygen transport in the brain: An efficient coarse-grid approach to capture perivascular gradients in the parenchyma
Source: PLoS Comput Biol. 2024 May 23;20(5):e1011973. doi: 10.1371/journal.pcbi.1011973 (PMC11257410; doi:10.1371/journal.pcbi.1011973)
Supplement: S1 Figures — (PDF) [file pcbi.1011973.s001.pdf]

Supplementary figures for "Modeling oxygen transport in the brain: an efficient coarse-grid approach to capture perivascular gradients in the parenchyma".

David Pastor-Alonso[1], Maxime Berg[1,2], Franck Boyer[3], Natalie Fomin-Thunemann[4], Michel Quintard[1], Yohan Davit[1], Sylvie Lorthois*[1],

1 Institut de Mécanique des Fluides de Toulouse, UMR 5502, CNRS, University of Toulouse, Toulouse, France.
2 Department of Mechanical Engineering, University College London, London, UK
3 Institut de Mathématiques de Toulouse (IMT), CNRS and Université de Toulouse, 31400 Toulouse, France
4 Department of Biomedical Engineering, Boston University, Boston, Massachusetts, USA.

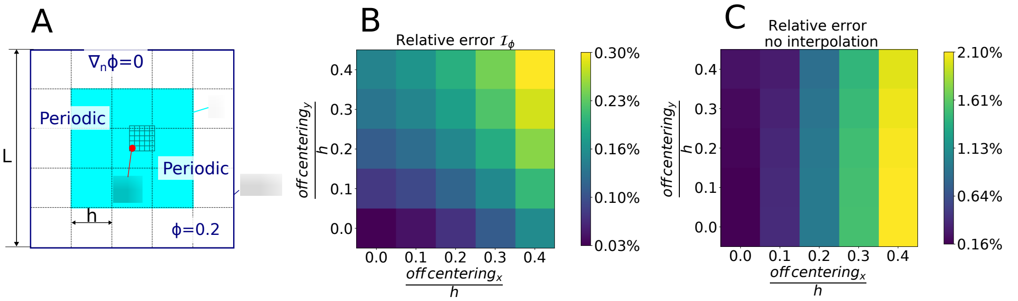

Fig A. Flux rate errors are displayed for the off-centering of a source withing a FV cell. A: schematics of the test-case where the center of the source is moved throughout the grid contained in the upper right corner of the center cell. B and C: error on the vessel-tissue exchanges ($\mathbf{q}$) with interpolation ($\mathscr{I}_\phi$) in Panel B and without in Panel C. The asymmetry of errors is due to the set of boundary conditions imposed at the limits of the computational domain (see Panel A).

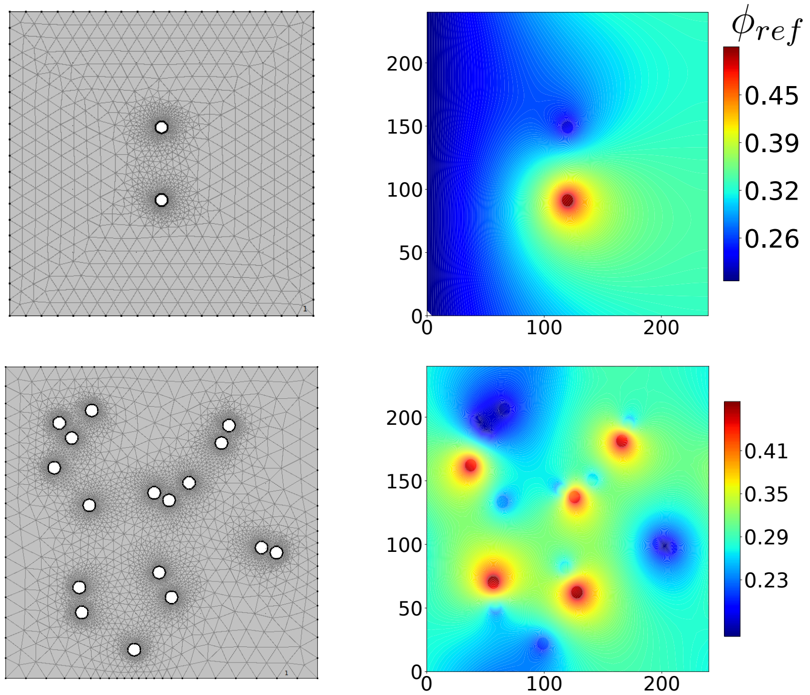

Fig B. Fine mesh finite element (FE) reference concentration field (ϕ_{ref}) obtained with COMSOL Multiphysics for the original non-reactive BVP (eqs. 1). For the single dipole configuration, 2278 mesh elements have been used (upper panel), while 9468 elements have been used for the multiple source configurations (lower panel).

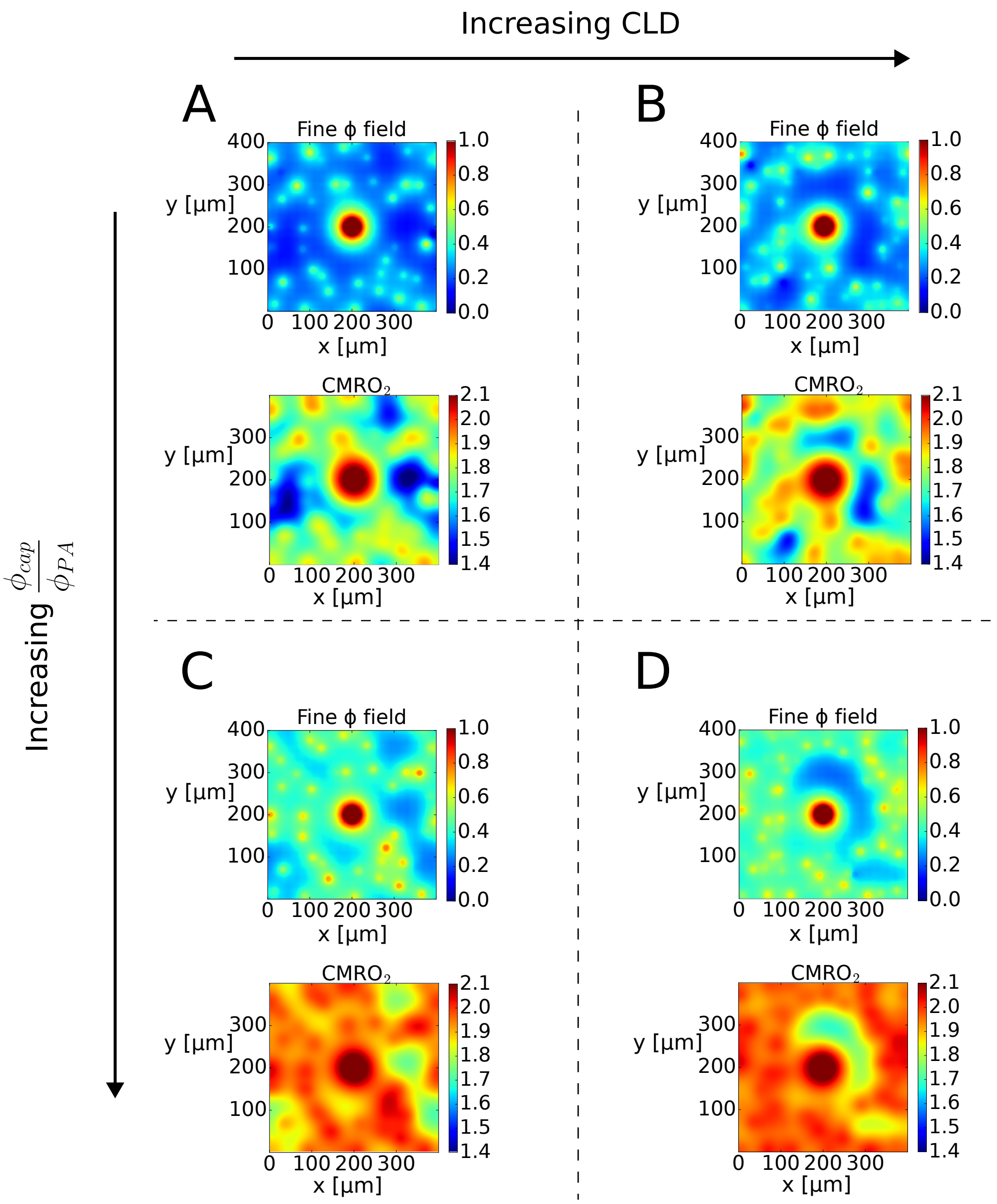

Fig C. Sub-grid reconstructions of the concentration field (upper) and local cerebral metabolic rate $MC/(K + C)$ (lower) for varying capillary densities and oxygenations. M=2.4 $\frac{\mu mol}{cm^3 min}$. A: CLD=0.8 mmm^{-3} and ϕ_{cap}/ϕ_{PA}=0.4; B: CLD=1.2 mmm^{-3} and ϕ_{cap}/ϕ_{PA}=0.4; C: CLD=0.8 mmm^{-3} and ϕ_{cap}/ϕ_{PA}=0.55; D: CLD=1.2 mmm^{-3} and ϕ_{cap}/ϕ_{PA}=0.55. To facilitate comparison, panels with same CLD present the same realization of source/sink locations.

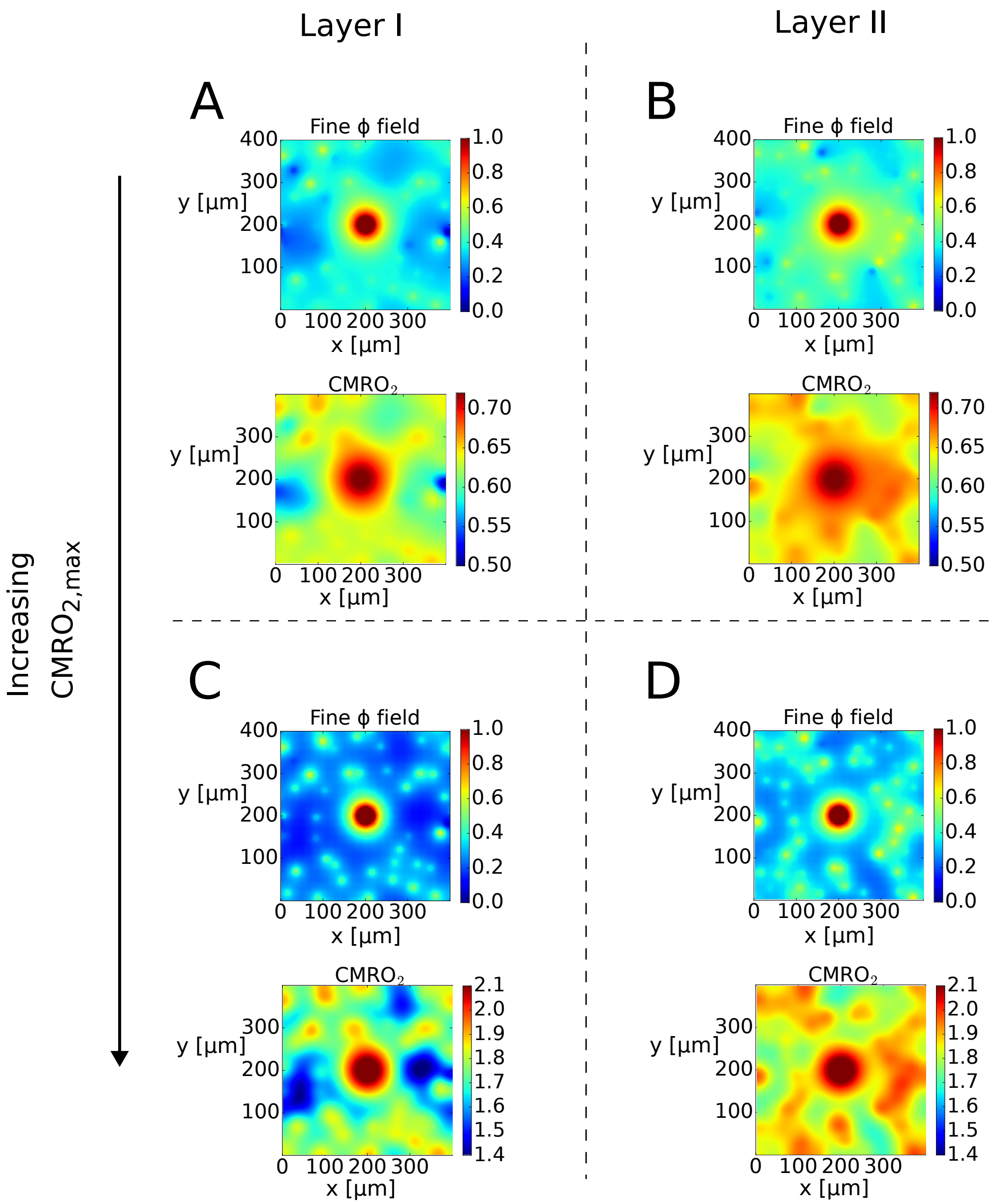

Fig D. Sub-grid reconstructions of the concentration field (upper) and local cerebral metabolic rate $MC/(K+C)$ (lower) for varying values of the maximal cerebral rate of oxygen M. Layer I: CLD=0.8 mmm^{-3} and ϕ_{cap}/ϕ_{PA}=0.4; Layer II: CLD=0.94 mmm^{-3} and ϕ_{cap}/ϕ_{PA}=0.45; A and B: M=0.8 $\frac{\mu mol}{cm^3 min}$; C and D: M=2.4 $\frac{\mu mol}{cm^3 min}$. To facilitate comparison, panels with same CLD present the same realization of source/sink locations.

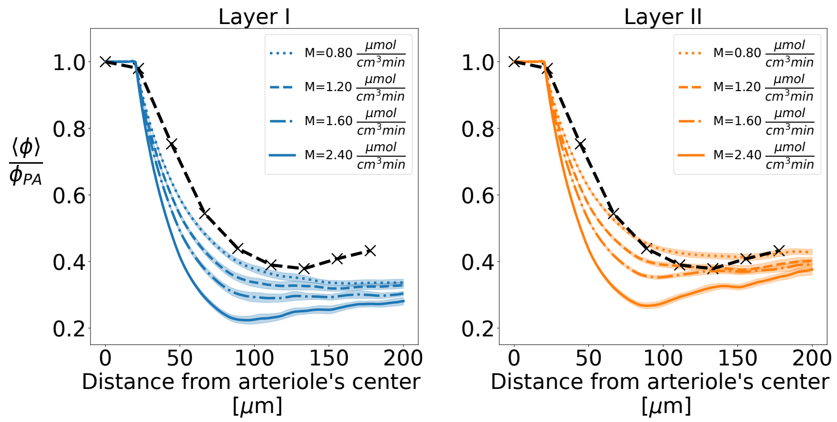

Fig E. Radial concentration profiles predicted in layer I and layer II (thin continuous lines) together with experimental values obtained from post-processing the data in Fig. 7C (bold dashed line). The predicted profiles are obtained by averaging the results of 30 simulations for parameters for layer I (left) and layer II (right) as given in Table 2, respectively, and for four physiologically realistic values of M (see legend in right panel). The experimental measurement were made $100 \mu m$ under the cortical surface, i.e., at the interface between layer I and II.
