# Supplementary material for: Modeling oxygen transport in the brain: An efficient coarse-grid approach to capture perivascular gradients in the parenchyma
Source: PLoS Comput Biol. 2024 May 23;20(5):e1011973. doi: 10.1371/journal.pcbi.1011973 (PMC11257410; doi:10.1371/journal.pcbi.1011973)
Supplement: S1 Methods — (PDF) [file pcbi.1011973.s002.pdf]

Supplementary methods for ”Modeling oxygen transport in the brain: an efficient coarse-grid approach to capture perivascular gradients in the parenchyma”.

David Pastor-Alonso[1], Maxime Berg[1,2], Franck Boyer[3], Natalie Fomin-Thunemann[4], Michel Quintard[1], Yohan Davit[1], Sylvie Lorthois*[1],

1 Institut de Mécanique des Fluides de Toulouse, UMR 5502, CNRS, University of Toulouse, Toulouse, France.
2 Department of Mechanical Engineering, University College London, London, UK
3 Institut de Mathématiques de Toulouse (IMT), CNRS and Université de Toulouse, 31400 Toulouse, France
4 Department of Biomedical Engineering, Boston University, Boston, Massachusetts, USA.

A Multiscale Finite Volume formulation

In the Finite Volume (FV) method, the main ideas are as follows:

1. **Discretization**: We divide the domain Ω_σ into a set of control volumes $\mathscr{F}$ where the variable are estimated at the geometrical center (node) of each control volume.
2. **Flux approximation**: We approximate the fluxes as a function of the nodal values to obtain a system involving only concentrations
3. **Closure of the system**: we impose flux and concentration continuity at each of the FV cell's interfaces

We begin by integrating the PDE 13a (see main text) over the control volume given by V_k:

$$\iint_{V_k} \nabla^2 \mathscr{s}_k(\mathbf{x}) dS = 0 \quad \forall k \in \mathscr{F} \tag{46}$$

Recalling that V_k have been introduced in Section 2.3.2 to provide a tessellation of the parenchyma Ω_σ, we obtain, by applying the divergence theorem:

$$\sum_{m \in \mathscr{N}^k} \int_{\partial V_{k,m}} (\boldsymbol{\nabla} \mathscr{s}_k \cdot \mathbf{n}) dl + \sum_{j \in E(V_k)} \oint_{\partial \Omega_{\beta,j}} (\boldsymbol{\nabla} \mathscr{s}_k \cdot \mathbf{n}) dl = 0 \quad \forall k \in \mathscr{F} \tag{47}$$

The second integral term in the left hand side of Eq. 47 is always null due to the boundary condition 13c. Using the TPFA (Eq. 15) to estimate the slow term gradients and the mid-point rule to evaluate the integrals, we obtain:

$$-4\widetilde{\mathscr{s}}_k + \sum_{m \in \mathscr{N}^k} \widetilde{\mathscr{s}}_{k,m} = 0 \tag{48}$$

with

$$\mathscr{N}^k := \{n, s, e, w\} \tag{49}$$

Since the goal is to obtain a system of equations with one value of the slow term for each FV cell, we need to remove the dummy variables of the slow term (i.e. $\widetilde{\mathscr{s}}_{k,m}$ and

$\widetilde{\mathscr{s}}_{m,k}$) at the interfaces of the FV cells. For that purpose, we use the local boundary conditions on each FV cell (Eqs 13d and 13e), which are rewritten below for ease of reading

$$
\begin{cases}
D\dfrac{\widetilde{\mathscr{s}}_{k,m} - \widetilde{\mathscr{s}}_k}{h/2} - D\dfrac{\widetilde{\mathscr{s}}_m - \widetilde{\mathscr{s}}_{m,k}}{h/2} = \dfrac{1}{h}\displaystyle\int_{\partial V_{k,m}} \mathbf{n} \cdot (D\boldsymbol{\nabla}\mathscr{r}_m(\mathbf{x}) - D\boldsymbol{\nabla}\mathscr{r}_k(\mathbf{x}))dl & \text{(50a)} \\
\widetilde{\mathscr{s}}_{k,m} - \widetilde{\mathscr{s}}_{m,k} = \dfrac{1}{h}\displaystyle\int_{\partial V_{k,m}} (\mathscr{r}_m - \mathscr{r}_k)dl & \text{(50b)}
\end{cases}
$$

We isolate $\widetilde{\mathscr{s}}_{m,k}$ in Eq. 50b and substitute it into Eq. 50 to obtain

$$
\widetilde{\mathscr{s}}_{k,m} = \frac{\widetilde{\mathscr{s}}_k + \widetilde{\mathscr{s}}_m}{2} + \frac{J_{k,m}}{2} \tag{51}
$$

where

$$
J_{k,m} = \frac{1}{2}\int_{\partial V_{k,m}} \mathbf{n} \cdot (\boldsymbol{\nabla}\mathscr{r}_m - \boldsymbol{\nabla}\mathscr{r}_k)dl + \frac{1}{h}\int_{\partial V_{k,m}} (\mathscr{r}_m - \mathscr{r}_k)dl \tag{52}
$$

Recalling that $\mathscr{r}_k$ and $\mathscr{r}_m$ are analytical functions of $\boldsymbol{q}$, we have written a system where the only unknowns are the values of the slow term in the FV grid ($\boldsymbol{\mathscr{s}}$) and the vessel-tissue exchanges ($\mathbf{q}$). Therefore, Eq. 16 results in

$$
-4\widetilde{\mathscr{s}}_k + \sum_{m \in \mathscr{N}^k} \left(\widetilde{\mathscr{s}}_m + J_{k,m}\right) = 0 \tag{53}
$$

where $J_{k,m} = J_{k,m}(\mathbf{q})$ since it only depends on the rapid term for each cell.

It is worth noting how Eq. 53 can be evaluated independently of the specific form of the rapid term. Additionally, the term $J_{k,m}$ is only evaluated at the interfaces of the cells V_k and V_m. This results in a more efficient and convenient formulation that allows for a flexible construction of the rapid term (see Section B) and a more efficient assembly of the system of equations compared to other models that impose a finite support to their respective analytical term [1, 2].

B Analytical derivation of potentials

To obtain Green's second identity, we multiply the concentration field in the parenchyma (ϕ) by a scalar field φ and apply the divergence theorem:

$$
\int_{\Omega_\sigma} \varphi\nabla^2\phi - \phi\nabla^2\varphi dV = -\oint_{\partial\Omega_\sigma} (\varphi\boldsymbol{\nabla}\phi - \phi\boldsymbol{\nabla}\varphi) \cdot \mathbf{n}dS \tag{54}
$$

where $\mathbf{n}$ follows the same convention as in figure 1 and represents the normal pointing inward to the domain Ω_σ, and $\partial\Omega_\sigma$ includes all the boundaries of the parenchyma (including the vascular walls) since $\partial\Omega_\sigma := \partial\Omega_\beta \bigcup \partial\Omega$.

We can obtain the Green's third identity by substituting the scalar field φ in equation 54 by the fundamental solution for the Laplace equation

$$
G(\mathbf{x}; \mathbf{x}^*) = \frac{1}{2\pi D}\ln\Big(\frac{a}{||\mathbf{x} - \mathbf{x}^*||}\Big) \tag{55}
$$

where a is a constant of integration. This yields

$$
D\nabla^2 G(\mathbf{x}; \mathbf{x}^*) = -\delta(\mathbf{x} - \mathbf{x}^*) \quad in \quad \Omega \tag{56}
$$

From equations 54 and 55,we obtain:

$$\phi(\mathbf{x}) = \oint_{\partial\Omega_\sigma} (\phi(\mathbf{x}^*)\boldsymbol{\nabla}G(\mathbf{x},\mathbf{x}^*)\cdot\mathbf{n}(\mathbf{x}^*) - G(\mathbf{x},\mathbf{x}^*)\boldsymbol{\nabla}\phi(\mathbf{x}^*)\cdot\mathbf{n}(\mathbf{x}^*))dS(\mathbf{x}^*) \tag{57}$$

which provides a description of the concentration field $\phi(\mathbf{x})$ through a superposition of a double layer potential and a single layer potential given as the first and second part of the integral, respectively. Note that here, the free space Green's function is used instead of the Green's function of the first or second kind due to the difficulty to calculate the later one. Practically, the approach used here is in line with the boundary element method literature [3–5]. Besides, we can decompose the integrals into a contribution from the external boundary $\partial\Omega$ and the vascular boundary $\partial\Omega_\beta$

$$\phi = \oint_{\partial\Omega} (\phi\boldsymbol{\nabla}G\cdot\mathbf{n} - G\boldsymbol{\nabla}\phi\cdot\mathbf{n})dl + \oint_{\partial\Omega_\beta} (\phi\boldsymbol{\nabla}G\cdot\mathbf{n} - G\boldsymbol{\nabla}\phi\cdot\mathbf{n})dl \tag{58}$$

where the integral over the boundary $\partial\Omega$ is expected to behave very regularly through space [2, 6–8] (*Assumption 1* in Section 2.6).

We now neglect the azimuthal variations of concentration around the sources (*Assumption 2* in Section 2.6). In 2D, this allows to strongly simplify the above expression since $\boldsymbol{\nabla}\phi\cdot\mathbf{n}$ and ϕ can be taken out of the integral. Then, the Green's function is integrated analytically over the source surface $\partial\Omega_{\beta,j}$

$$\oint_{\partial\Omega_{\beta,j}} G(\mathbf{x};\mathbf{x}^*)d\mathbf{x}^* = \begin{cases} R_j \ln\Big(\dfrac{R_j}{||\mathbf{x}-\mathbf{x}_j||}\Big) + K_1 & \text{if } ||\mathbf{x}-\mathbf{x}_j|| > R_j \\ K_1 & \text{if } ||\mathbf{x}-\mathbf{x}_j|| \leq R_j \end{cases} \tag{59}$$

where $\mathbf{x}_j$ is the center of the circular source ($\Omega_{\beta,j}$) and K_1 is a constant arising from the integration. Furthermore, due to the simplified Robin boundary condition (Eq. 2) we know that, on the outer surface of the source,

$$-\mathbf{n}\cdot(\boldsymbol{\nabla}\phi) = \frac{q_j}{2\pi R_j D} \tag{60}$$

Therefore, we obtain a non-integral expression for the single layer potential of each source as a function of the vessel-tissue exchanges (q_j)

$$-\oint_{\partial\Omega_{\beta,j}} (G(\mathbf{x};\mathbf{x}^*)\boldsymbol{\nabla}\phi(\mathbf{x}^*))\cdot\mathbf{n}(\mathbf{x}^*)d\mathbf{x}^* = \frac{q_j}{2\pi D}\ln\Big(\frac{R_j}{||\mathbf{x}-\mathbf{x}_j||}\Big) + K_1 \text{ for } \mathbf{x}\in\Omega_\sigma \tag{61}$$

For convenience we set $K_1 = \overline{\phi}_j$ so the potential always stays positive. Furthermore, since the sources in a 2D simulation are closed surfaces, the double layer potential is:

$$\oint_{\partial\Omega_{\beta,j}} (\boldsymbol{\nabla}G(\mathbf{x};\mathbf{x}^*)\cdot\mathbf{n}(\mathbf{x}^*))d\mathbf{x}^* = 0 \quad \forall\mathbf{x}\notin\Omega_{\beta,j} \tag{62}$$

Thus, Eq. 58 simplifies to:

$$\phi(\mathbf{x}) = \oint_{\partial\Omega} (\phi\boldsymbol{\nabla}G\cdot\mathbf{n} - G\boldsymbol{\nabla}\phi\cdot\mathbf{n})dl + \sum_{j\in E(\Omega)} P_j \tag{63}$$

with

$$P_j = \overline{\phi}_j + \frac{q_j}{2\pi D}\ln\Big(\frac{R_j}{||\mathbf{x}-\mathbf{x}_j||}\Big) \quad \text{if } ||\mathbf{x}-\mathbf{x}_j|| > R_j \ \forall\mathbf{x}\in\Omega_\sigma \tag{64}$$

We now make the link between the Green's formulation and the field splitting introduced in Section 2.2. When the neighbourhood of influence of each source is the whole domain ($\widehat{V}_k = \Omega \quad \forall k \in \mathscr{F}$)

$$\phi(\mathbf{x}) = \mathscr{s}(\mathbf{x}) + \sum_{j \in E(\Omega)} \Big(\overline{\phi}_j + \frac{q_j}{2\pi D} \ln\Big(\frac{R_j}{||\mathbf{x}-\mathbf{x}_j||} \Big) \Big) \tag{65}$$

Therefore,

$$\mathscr{s}(\mathbf{x}) = \oint_{\partial\Omega} (\phi \boldsymbol{\nabla} G \cdot \mathbf{n} - G \boldsymbol{\nabla} \phi \cdot \mathbf{n}) dl \tag{66}$$

demonstrating that the slow term carries the contribution of the boundary conditions.

We can reduce the size of $\widehat{V}$ to localize the potentials and increase the sparseness of the system, as the slow term then compensates for the contribution of sources lying further away. Essentially, when we decrease the size of $\widehat{V}$, we rely on the low gradients of the potentials far away from the sources to be accommodated by the slow term. In other words, when we decrease the size of $\widehat{V}$, the number of sources modeled analytically decreases and the gradient of the slow term increases (see Section 2.3.4 for an estimation of the numerical errors as a function of the the size of $\widehat{V}$, n). Nevertheless, the slow term behaves very regularly under most circumstances [1, 6, 9].

C Model conservativeness

Due to the approximated form for the source potentials given in Eq. 24, Eq. 9b is not satisfied point-wise when multiple sources lie close together. For ease of readability we recall the definition of the rapid term (Eq. 9):

$$\begin{cases} \nabla^2 \mathscr{r}_k = 0 & \text{in } \ \Omega_\sigma & (67a) \\ -\mathbf{n} \cdot (D \boldsymbol{\nabla} \mathscr{r}_k) = \dfrac{q_j}{2\pi R_j} & \text{on } \ \partial\Omega_{\beta,j} \, \forall j \in E(V_k) & (67b) \end{cases}$$

where $\mathscr{r}_k$ is composed by the linear sum of the sources potentials in the neighbhourhood (see Section 2.3.2 and B):

$$\mathscr{r}_k = \sum_{j \in E(\widehat{V}_k)} P_j \tag{68}$$

and the potential for each source is given by

$$P_j = \begin{cases} \overline{\phi}_j + \dfrac{q_j}{2\pi D} \ln\Big(\dfrac{R_j}{||\mathbf{x}-\mathbf{x}_j||} \Big) & \text{if } ||\mathbf{x}-\mathbf{x}_j|| > R_j \\ \overline{\phi}_j & \text{if } ||\mathbf{x}-\mathbf{x}_j|| \leq R_j \end{cases} \tag{69}$$

As a result of the choice of P_j, when two sources lie close together, Eq. 67b is not strictly satisfied anymore. To illustrate this, let us suppose there are two sources in the domain, both lying within the same mesh cell k i.e.$E(\Omega) = E(V_k) = \{1, 2\}$,. The rapid term reads

$$\mathscr{r}_k = P_1 + P_2 \tag{70}$$

We then evaluate Eq. 67b

$$\begin{cases} -\mathbf{n} \cdot (D \boldsymbol{\nabla} \mathscr{r}_k)|_{\partial\Omega_{\beta_1}} = \dfrac{q_1}{2\pi R_j} + \varepsilon^q_{2,1}(\mathbf{x}; q_2) & (71a) \\ -\mathbf{n} \cdot (D \boldsymbol{\nabla} \mathscr{r}_k)|_{\partial\Omega_{\beta_2}} = \dfrac{q_2}{2\pi R_j} + \varepsilon^q_{1,2}(\mathbf{x}; q_1) & (71b) \end{cases}$$

where $\varepsilon_{i,j}$ is the error caused by the potential of source i on source j, which stays undefined since we have not specified the position of each source. We can generalize the case for multiple sources:

$$-\mathbf{n} \cdot (D\boldsymbol{\nabla}\mathscr{r}_k) = \frac{q_i}{2\pi R_i} + \sum_{j\in E(\widehat{V}_k), j\neq i} \varepsilon^q_{i,j} \quad \text{on } \partial\Omega_{\beta,i} \quad \forall i \in E(V_k) \tag{72}$$

and

$$-\mathbf{n} \cdot (D\boldsymbol{\nabla}\mathscr{s}_k) = \sum_{j\in E(\widehat{V}_k), j\neq i} \varepsilon^q_{i,j} \quad \text{on } \partial\Omega_{\beta,i} \quad \forall i \in E(V_k) \tag{73}$$

However, as P_j are harmonic functions (Eq. 24), applying the divergence theorem we see that the integral contribution of these perturbations around source i is null

$$\oint_{\partial\Omega_{\beta,i}} \varepsilon^q_{i,j} dl = 0 \quad \text{for } i \neq j \tag{74}$$

ensuring that the model remains conservative. Therefore, we can conclude that Eq. 9 may not be satisfied point-wise, but the integral contribution of the associted error is null.

D Sub-grid interpolation of the concentration field

The purpose of the interpolation scheme is two-fold. Firstly, we need to estimate the wall concentration $\overline{\phi}$ to evaluate Eq. 3, and secondly, we aim at providing a sub-grid reconstruction of the concentration field from the values obtained at the grid nodes

$$\widetilde{\phi}_k = \widetilde{\mathscr{s}}_k + \mathscr{r}_k(\mathbf{x}_k) \quad \forall k \in \mathscr{T} \tag{75}$$

where $\mathscr{T}$ represents the set of all grid nodes $\mathscr{F}$ plus the boundary nodes, and $\mathbf{x}_k$ represents the position of each node. We want to preserve the logarithmic nature of the source potentials, therefore we define a dual neighbourhood $\widehat{V}^d$ that defines the sources whose potentials are reconstructed analytically. We use linear shape functions to interpolate the node values of the slow term. Since there are discontinuities across the mesh cell faces, a correction term $\mathbb{C}_i$ is added to preserve continuity of the interpolated field. We thus obtain:

$$\mathscr{I}_\phi(\mathbf{x}) = \sum_{i\in\mathscr{T}} \gamma_i(\mathbf{x})(\widetilde{\mathscr{s}}_i + \mathbb{C}_i) + \sum_{j\in E(\widehat{V}^d)} P_j(\mathbf{x}) \tag{76}$$

where $\mathscr{I}_\phi$ is the interpolation function and γ_i are the classic linear shape functions for a square element [5].

The choice of $\widehat{V}^d$ is arbitrary. In our case, we choose the union of the neighbourhoods ($\widehat{V}$) of all FV cells involved in the interpolation. Therefore, following the convention in Fig. 2, inside the red shaded space, we suggest:

$$\widehat{V}^d = \bigcup_{i\in\{k,e,s,se\}} \widehat{V}_i \tag{77}$$

To estimate the correction $\mathbb{C}_i$, we impose the constraint that:

$$\mathscr{I}_\phi(\mathbf{x}_k) = \widetilde{\phi}_k \quad \forall k \in \mathscr{T} \tag{78}$$

We further use Eqs. 78, 75 and 76 to solve for $\mathbb{C}_i$:

$$\mathbb{C}_i = -\sum_{j\in E(\widehat{V}^d)} \delta_{ji} P_j(\mathbf{x}_i) \tag{79}$$

where

$$\delta_{ji} = \begin{cases} 0 & \text{if} \quad j \in \widehat{V}_i \\ 1 & \text{else} \end{cases} \tag{80}$$

For ease of readability, we group all the potential terms into a extended rapid term:

$$\mathscr{r}_i^c(\mathbf{x}) = \sum_{j \in E(\widehat{V}^d)} (P_j(\mathbf{x}) - \delta_{ji} P_j(\mathbf{x}_i)) \tag{81}$$

to finally obtain the interpolation function

$$\mathscr{I}_\phi(\mathbf{x}) = \sum_{i \in \mathscr{T}} \gamma_i(\mathbf{x}) (\widetilde{\mathfrak{z}}_i + \mathscr{r}_i^c(\mathbf{x})) \tag{82}$$

E Metabolism

As written in section 2.4, the integrals inside vector $\mathbf{S_{metab}}$ are evaluated using the Simpson's rule of integration

$$\mathbf{S_{metab}} = \begin{Bmatrix} \frac{M}{D}\left(1 - Si_{V_1}\left(\frac{\phi_0}{\phi_0 + \widetilde{\mathfrak{z}}_1 + \mathscr{r}_1(\mathbf{x})}\right)\right) \\ \frac{M}{D}\left(1 - Si_{V_2}\left(\frac{\phi_0}{\phi_0 + \widetilde{\mathfrak{z}}_2 + \mathscr{r}_2(\mathbf{x})}\right)\right) \\ \frac{M}{D}\left(1 - Si_{V_2}\left(\frac{\phi_0}{\phi_0 + \widetilde{\mathfrak{z}}_3 + \mathscr{r}_3(\mathbf{x})}\right)\right) \\ \vdots \\ \frac{M}{D}\left(1 - Si_{V_F}\left(\frac{\phi_0}{\phi_0 + \widetilde{\mathfrak{z}}_F + \mathscr{r}_F(\mathbf{x})}\right)\right) \end{Bmatrix} \tag{83}$$

where Si refers to the second order accurate Simpson's rule of integration. We have the following system of equations for the iterative system

$$J(\boldsymbol{\mathfrak{s}}^n, \mathbf{q}^n) \begin{Bmatrix} \Delta \boldsymbol{\mathfrak{s}} \\ \Delta \mathbf{q} \end{Bmatrix} = - \begin{bmatrix} \mathsf{A} & \mathsf{B} \\ \mathsf{C} & \mathsf{E} \end{bmatrix} \cdot \begin{Bmatrix} \boldsymbol{\mathfrak{s}}^n \\ \mathbf{q}^n \end{Bmatrix} - \begin{Bmatrix} \mathbf{b}_{\partial\boldsymbol{\Omega}} \\ \mathbf{b}_{\partial\boldsymbol{\Omega}_\beta} \end{Bmatrix} \tag{84}$$

where each new iteration is given by

$$\begin{Bmatrix} \boldsymbol{\mathfrak{s}}^{n+1} \\ \mathbf{q}^{n+1} \end{Bmatrix} = \begin{Bmatrix} \boldsymbol{\mathfrak{s}}^n \\ \mathbf{q}^n \end{Bmatrix} + \begin{Bmatrix} \Delta \boldsymbol{\mathfrak{s}} \\ \Delta \mathbf{q} \end{Bmatrix} \tag{85}$$

and the Jacobian is calculated as

$$J(\boldsymbol{\mathfrak{s}}^n, \mathbf{q}^n) = \begin{bmatrix} \mathsf{A} & \mathsf{B} \\ \mathsf{C} & \mathsf{E} \end{bmatrix} + \begin{bmatrix} \frac{\partial \mathbf{S_{metab}}}{\partial \boldsymbol{\mathfrak{s}}^n} & \frac{\partial \mathbf{S_{metab}}}{\partial \mathbf{q}^n} \\ \mathbf{0} & \mathbf{0} \end{bmatrix} \tag{86}$$

$$\left[\frac{\partial \mathbf{S_{metab}}}{\partial \boldsymbol{\mathfrak{s}}^n}\right]_{k,m} = \begin{cases} 0 & \text{if } k \neq m \\ -\frac{M}{D} Si_{V_k}\left(\frac{\phi_0}{(\phi_0 + \widetilde{\mathfrak{z}}_k + \mathscr{r}_k(\mathbf{x}))^2}\right)) & \text{if } k = m \end{cases} \tag{87}$$

$$\left[\frac{\partial \mathbf{S_{metab}}}{\partial \mathbf{q}^n}\right]_{k,j} = \begin{cases} 0 & \text{if } j \notin E(\widehat{V}_k) \\ -\frac{M}{D} Si_{V_k}\left(\frac{1}{2\pi R_j} \ln\left(\frac{R_j}{||\mathbf{x} - \mathbf{x}_j||}\right) \frac{\phi_0}{(\phi_0 + \widetilde{\mathfrak{z}}_k + \mathscr{r}_k(\mathbf{x}))^2}\right) & \text{if } j \in E(\widehat{V}_k) \end{cases} \tag{88}$$

The initial guess for the iterative system is the solution for the linear system with no metabolism

$$\begin{bmatrix} \mathsf{A} & \mathsf{B} \\ \mathsf{C} & \mathsf{E} \end{bmatrix} \cdot \begin{Bmatrix} \boldsymbol{\mathcal{s}}^0 \\ \mathbf{q}^0 \end{Bmatrix} = \begin{Bmatrix} \mathbf{b_1} \\ \mathbf{b_2} \end{Bmatrix} \tag{89}$$

References

1. Gjerde IG, Kumar K, Nordbotten JM. A singularity removal method for coupled 1D–3D flow models. Computational Geosciences. 2020;24(2):443–457. doi:10.1007/s10596-019-09899-4.

2. Wolfsteiner C, Lee SH, Tchelepi HA. Well Modeling in the Multiscale Finite Volume Method for Subsurface Flow Simulation. Multiscale Modeling & Simulation. 2006;5(3):900–917. doi:10.1137/050640771.

3. Pozrikidis C, Farrow DA. A Model of Fluid Flow in Solid Tumors. Annals of Biomedical Engineering. 2003;31(2):181–194. doi:10.1114/1.1540103.

4. Aliabadi MH, Wen PH. Boundary element methods in engineering and sciences. No. 4 in Computational and experimental methods in structures. London: Imperial College Press; 2011.

5. Pepper D, Kassab A, Divo E. Introduction to Finite Element, Boundary Element, and Meshless Methods: With Applications to Heat Transfer and Fluid Flow. ASME Press; 2014.

6. Roach GF. Green's Functions. 2nd ed. Cambridge: Cambridge University Press; 1982.

7. Pozrikidis C, Davis JM. Blood Flow Through Capillary Networks. In: Transport in Biological Media. Elsevier; 2013. p. 213–252.

8. Ding DY. Near-Well Upscaling for Reservoir Simulations. Oil & Gas Science and Technology. 2004;59(2):157–165. doi:10/dpvbh3.

9. Pozrikidis C, Ferziger JH. Introduction to Theoretical and Computational Fluid Dynamics. Physics Today. 1997;50(9):72–74. doi:10.1063/1.881920.
