# Supplementary material for: Modeling oxygen transport in the brain: An efficient coarse-grid approach to capture perivascular gradients in the parenchyma
Source: PLoS Comput Biol. 2024 May 23;20(5):e1011973. doi: 10.1371/journal.pcbi.1011973 (PMC11257410; doi:10.1371/journal.pcbi.1011973)
Supplement: Attachment — Submitted filename: Response_to_reviewers_V6.pdf [file pcbi.1011973.s003.pdf]

Response to reviewer's comment on manuscript "Modeling oxygen transport in the brain: An efficient coarse-grid approach to capture perivascular gradients in the parenchyma"

We sincerely thank the reviewers for their constructive comments. Below, we provide answers to all their comments and summarize the changes made to our manuscript. Please note that all references, line, page and figure numbers below refer to the attached revised version tracking changes from the first submission (in blue in the text). In the same way, all references to colors or line-styles in figures refer to conventions adopted in this revised version. Please note that, in addition to changes requested by these reviewers, we also took the opportunity to correct for a few typos and rephrase a one paragraph to improve clarity (i.e. lines 436 to 444). These additional changes are also highlighted in blue in the revised version. Please also note that Supplementary Information files (without and with revision marks) have been provided separately at the request of the editorial team, and can be now accessed by clicking on the links on p 54 and p55 of the assembled submission package, respectively.

Responses to Reviewer 1:

We sincerely thank this reviewer for its faithful synthesis of the contributions presented in our manuscript and his positive general conclusions about the novel numerical approach for solving oxygen transport in the brain parenchyma.

Specific comments:

Comment 1. *"Line 72: The limitations of standard numerical methods (finite difference, finite element, etc.) are well addressed in this section. However, the capabilities and limitations of the well-established Green's-function approach should also be discussed in this section. (See for example Celaya-Alcala JT et al., Simulation of oxygen transport and estimation of tissue perfusion in extensive microvascular networks: Application to cerebral cortex. J Cereb Blood Flow Metab 41: 656-669, 2021.) The key point in this context is that the Green's-function approach requires solving a large non-sparse system."*

Response: We agree with Reviewer 1 that the well-established Green's-function approach should have been discussed in the Introduction Section and that the key point is that it requires solving a large non-sparse system, even if other additional drawbacks are also addressed by our approach, including the easiest and more versatile implementation of boundary conditions and non-linear metabolic reactions.

Changes made: Accordingly, we have thus added the following paragraph at line 77, which also gave the opportunity to add the suggested reference:

"A popular alternative, specifically designed to solve oxygen transport in the microcirculation, formulates the problem using Green's functions [31-35]. The non-local nature of this formulation allows the description of concentration gradients around microvessels while circumventing the need for meshing the intricate geometry of the extravascular space. However, it generally relies on the infinite domain form of the Green's function, making difficult the application of boundary conditions at the limits of the tissue domain (e.g. periodic boundary conditions). Additionally, oxygen metabolism exhibits non-linear behavior [23,36], which is challenging to describe using Green's function and generally requires additional meshing [31-37]. This, coupled with the non-local formulation at the core of the approach, requires the creation of large and dense matrices that are computationally costly to invert."

To insist on key point mentioned by this referee, we also added the adjective "non-sparse" at line 279.

Comment 2. *"Line 201: for harmonic functions such as s(x), from Gauss's harmonic function theorem [54], …, this average value is equal to the value at the cell's center." This is not true for non-circular domains. For example, consider the function cosh(pi x) cos(pi y) on the unit square centered on the origin. The average on the domain is 4 sinh(pi/2)/pi^2, which is 0.932, but the value at the center is 1. At best, this must be considered as an approximation."*

Response: We thank the reviewer for pointing out this error. Indeed, Gauss harmonic function theorem is only true for circular domains. For square domains, it only constitutes an approximation. However, it can be shown it is a second-order approximation.

Gauss harmonic function theorem indeed straightforwardly generalizes to close contours of any shape for constant functions, and to contours exhibiting central symmetry around their barycenter, including squares and rectangles, for linear functions. Using Taylor's expansion, we can conclude that only second-order terms remain in the approximation error, which we consider acceptable for the context of oxygen transport in the brain parenchyma.

Changes made: The above sentence has thus been reformulated as follows in the revised MS (see lines 216-217).

"For harmonic functions such as s(x), from Gauss's harmonic function theorem [54], …, this average value can be approximated at second-order by the value at the cell's center".

Comment 3. *"Line 273: The presentation of the model is 2D, with prescribed oxygen levels in vessels, and the coupling of diffusive and convective transport is not addressed. It is stated that "The rest of the model presented in section 2.3 can be simply extrapolated to 3D." However, a proper 3D treatment would require modeling convection in blood vessels, which is not a simple extension.""*

Response: We agree with Reviewer 1 that a proper 3D treatment would require modeling convective transport within blood vessels in addition to diffusive transport in the parenchyma. We preferred discussing this coupling and the associated methodological aspects in the Discussion (lines 667-690 of the original submission, lines 719-732 of the revised version), rather than in the Model and Methods Section. However, we understand that the formulation we used and underlined by Reviewer 1 was misleading.

Changes made: Consistently, to make clearer that the simple extrapolation we were referring to in this Section only concerns the developments presented in Section 2.3, we reformulated the above sentence as follows (see line 289-291 of the revised version):

"The rest of the ***developments*** *presented in section 2.3,* ***including FV discretization and localization of the slow term,*** *can be simply extrapolated to 3D."*

Minor comments

1. P. 1, abstract last sentence, also line 737, should be "reconciling", line 617 should be "reconcile"

Many thanks for pointing this typo out. We have corrected as suggested.

2. Line 59, "breakthroughs"

We have changed the grammatical form to plural.

3. Line 142, "lineic" is not a word. Say "per unit length"

This has been corrected.

4. Line 630: "capture"

This has been corrected.

5. Lines 735 and 738, "periarteriolar"

This has been corrected.

6. P. 37, ref. 39 is incomplete

Ref 39 has been completed. Many thanks for pointing out to this incomplete reference.

Responses to Reviewer 2:

We thank Reviewer 2 for his constructive comments, that will offer opportunities to clarify the presentation of our work. Before providing answers to all his comments and summarizing the changes made to our manuscript, we wish to underline that, as written in Section "Code Availability", all author-generated python code, as well as Comsol reference simulation data used for comparison, had been included as Supplementary Material with our initial submission (see Code Availability, lines 784-788) . We regret access to this material seemed to be not straightforward through the Editorial system, and agree that interested readers could find additional details in the source code that may be helpful. We reattach this same Supplementary Material to the present submission, and reaffirm our commitment to upload this material on a public repository after acceptance.

Comment 1: *"The main issue with the work submitted is that a big portion of the goal is to solve the inverse problem of determining metabolic rates, this part remains a little weak and unclear in that there is no direct simulation to determine errors based on metabolic rates."*

Response: We agree with Reviewer 2 that solving the inverse problem of determining metabolic rates in the brain would be an important achievement. In line with this comment, we emphasized in the Abstract (end of first paragraph) and in the Introduction (see lines 88-93) that one of the main bottlenecks toward this end is the heavy computational requirements of existing direct methods for simulating oxygen transport and consumption within the brain parenchyma. Thus, our goal in this manuscript was to introduce a novel approach for that purpose that considerably reduces the computational cost while maintaining accuracy, as written in the introductory paragraph describing the MS goals (lines 113-115).

Changes made: To clarify ambiguities on the MS goals, we reformulated the first sentence of this paragraph as follows: "*The goal of the present paper is to revisit the problem of oxygen transport and metabolism in the brain parenchyma to introduce a simple, scalable and accurate numerical method for its **direct** resolution.*" (addition of adjective "*direct*", see line (115). We also added the word "*direct*" in the Abstract (first paragraph) and in the Discussion (line 665).

Comment 2: "This is likely embedded in the sources and sinks portion of the work (a sink presumably being a large metabolically active unit and a source presumably a penetrating artery with high oxygen), but this presentation is not sufficiently clear."

Response: We thank Reviewer 2 for pointing out the misleading terminology associated to the use of "*sources*" and "*sinks*" in our manuscript. While metabolically active units indeed consume oxygen and could be designated by "sinks", we instead chose to differentiate between vessels that locally supply oxygen to the tissue, typically arterioles and post-arteriolar capillaries, and vessels that locally reabsorb oxygen, typically venules and pre-venular capillaries. Oxygen reabsorption by venules has indeed been evidenced experimentally, resulting in "diffusional shunts" between arterioles and venules through the parenchyma (see e.g. Lecoq et al. 2011[1]), that we wanted our model to represent accurately. We referred to the first category of vessels by "*sources*" and to the second category by "*sinks*".

From a quantitative point of view, the only difference between sources and sinks is the sign of the integral molecular flux through the vessel wall q_j. In general, this sign is not known a priori but results from the coupling between intravascular transport, transport through the vessel wall and transport and consumption in the parenchyma. However, in this paper we focus on idealized cases, where a subset of vessels are all assigned a concentration equal to zero, and the remaining of vessels are all assigned an equal, strictly positive, concentration (e.g. of unit value). In such cases, the sign of all *qj*'s is known a priori. This sign is negative for the first subset, that we then designate as sinks. It is positive for the second subset, that we then designate as sources[2].

[1] Lecoq, J. et al. Simultaneous two-photon imaging of oxygen and blood flow in deep cerebral vessels. Nature Medicine 17, 893–898 (2011).

[2] In the non-reactive case, this directly results from the mathematical properties of harmonic functions, the maxima and minima of which must be located at the frontier of the considered domain. Oxygen concentrations in the tissue are thus comprised between the imposed sink and source concentration, setting up the signs of all qj's through Equation 27. In the reactive case, interaction with consumption could in general result in tissue concentrations that become locally lower than the lowest vessel concentration. However, this is not the case for Michaelis-Menten kinetics if the lowest vessel concentration is chosen to be null, as then there is locally vanishing tissue consumption.

Changes made: In order to improve clarity of the Manuscript with regard to the above comment, we have clarified the meaning of “source” and “sink” by adding the following sentence at the beginning of Section 3 Results: error estimation (see lines 392-396 of the revised MS):

“*Noteworthy, we generically designate by “source” any vessel* j *whose concentration is greater than the local tissue concentration, i.e., for which the resulting flux* q_j *will be positive. In the same way, we use “sink” for any vessel* j *whose concentration is lower than the local tissue concentration, i.e., for which the resulting flux* q_j *will be negative*.”

Comment 3: *“Another potential issue is that the work ends without testing for regimes or manipulations where simpler models could do well enough at estimating metabolic rates; for example, if oxygen supply is doubled but M remains the same or if oxygen supply is halved and M remains the same. It would be a novel contribution to describe conditions or manipulations that would help mitigate errors from either standard model vs. this model.”*

Response: We agree with Reviewer 2 that we do not provide a systematic parametric analysis that explicitely discriminates between regimes where the present model is required and regimes where standard models could be sufficient. However, with this regard, it is important to underline that neither the standard models, based on the Krogh configuration (e.g. ref 24 in our MS), nor the recent ODACITI improvement (ref 10 in our MS) that relaxes the assumption that the capillary-free zone around arterioles is not supplied by more distant capillaries, enable to integrate a priori knowledge on the oxygenation of the capillary bed or on the capillary density for the estimation of *M*. As a result, the sensitivity of the oxygen gradient in the vicinity of a penetrating arteriole (perivascular oxygen gradient, POG) only depends on some geometrical parameters (e.g. arteriolar radius, size of capillary-free zone) and on the value of *M.*

In contrast, with fixed values of these geometrical parameters, the direct simulations presented in Section 4 demonstrate that the strength of the oxygen gradient in the vicinity of a penetrating arteriole is not only sensitive to *M* but also to the capillary density and oxygenation in the (more distant) brain tissue. To roughly assess these sensitivities, we can compare simulations presented in Figure 7E (where *M* is constant and equal to 2.4 μM/cm^3/min but capillary density increases by 50% and capillary oxygen, normalized by oxygen in the arteriole, increases by 37,5% from layer I to layer IV) and simulations presented in Fig. S5, left (where capillary density and oxygenation are constant and equal to their values in layer I but *M* is more than halved). These show that, starting from the parameters in layer I (M=2.4 μM/cm^3/min, CLD=0.8 m/mm^3, $\frac{\varphi_{cap}}{\varphi_{art}}$=0,4), increasing the capillary density and oxygenation by 50% and 37,5%, respectively (ie. from layer I to layer IV, or from continuous blue to red line in Fig 7E) has a similar impact on the POGs than decreasing *M* by a factor 1/3: in fact, the red curve in the main panel of fig 7E (M=2.4 μM/cm^3/min, CLD=1.2 m/mm^3, $\frac{\varphi_{cap}}{\varphi_{art}}$=0,55) has a similar periarteriolar dynamics as the dashed-dotted blue curve in Fig S5 (initially Fig S3), left (M=1.6 μM/cm^3/min, CLD=0.8 m/mm^3, $\frac{\varphi_{cap}}{\varphi_{art}}$=0,4). These three curves are integrated below in the same figure for facilitating comparison.

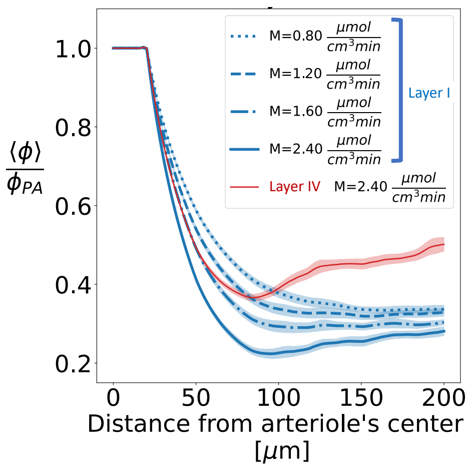

Overall, this shows that variations in capillary density and oxygenation should be taken into account to infer *M* from POGs across cortical layers.

Of course, a systematic analysis of the behavior of our direct model as a function of the above parameters could be performed. However, because of the strong coupling between capillary oxygenation and blood flow, such a

parametric analysis should integrate intravascular transport to be relevant from a physiological point of view, as was shortly discussed in our manuscript (lines 719-732). Thus, in the present paper, we limited our parametric analysis to focus on the impact of variations across layers. However, to account for the present comment, we have enriched our manuscript by adding supplementary figures (Fig S3 and S4) that display the spatial distributions of concentrations and local metabolic rate for relevant combinations of parameters.

Changes made: In order to better highlight the sensitivity of POGs to *M* and capillary density and oxygenation, we added two supplementary figures. Supplementary figure S3 displays the spatial distribution of oxygen concentration and local metabolic rate (*MC/(K+C))* if *M* remains the same but capillary density increases by 50% and/or capillary oxygen increases by 37,5% (similar to variations from layer I to layer IV). Supplementary figure S4 displays the spatial distribution of oxygen concentration and local metabolic rate if capillary density and oxygenation remain the same but *M* is more than halved. Further, to improve readability and comparison between Fig 7E and Fig S5 (initially Fig S3), we have adopted more consistent conventions for colors and line-styles in these figures, with a single color corresponding to a single combination of capillary density/oxygenation and a single line-style for a single value of *M*. Finally, we have enriched the text in Section 4 to better highlight that the sensitivity of POGs to these physiologically realistic variations of *M* is of the same order than to realistic variations of capillary density and oxygenation (see lines 617-649).

Comment 4: "It would be useful to see results at various capillary densities with the same input (flow and oxygen) and same metabolic rate, since different cortical layers have different densities (acknowledged by the authors) but these results are not systemically presented."

Response: We agree with Reviewer 2 that *seeing* the heterogeneity of the concentration at various capillaries densities with same input can be useful to the reader. In the initial version of our manuscript, we had only provided integral parameters, such as the average concentration in the capillary-free zone, over many realizations, for various capillary densities and oxygen concentrations in the capillaries, but the same maximal metabolic rate of oxygen (Figure 7F). To complement the analysis, we have thus added relevant corresponding concentration and local metabolic rate snapshots as supplementary figure S4, as explained in our answer to Comment 3.

However, we want to clarify that, in its current version, our model does not include a description of cerebral blood flow. To this end, extension to 3D and coupling with intravascular transport is required, which presents no strong conceptual difficulties, as shortly discussed in our manuscript (see lines 719-732). However, even in 2D, the introduced conceptual framework is rather complex, as noted by both reviewers, so that we chose to develop the model in 2D "to increase the readability of the mathematical developments and to enable exploitation of current commercial finite element solvers" for validation, as written in the Introduction (lines 122-125). That is why we argue that extension to 3D is out of the scope of this paper.

Changes made: In response to this and the previous comment, Fig S4 has been added.

Comment 5: Have the authors attempted to model the more superficial layer where the oxygen tensions are relatively high? Especially in Layer 1 where most processes exist vs. Layer 2 or deeper layers? This likely sets up other difficulties but it would be interesting to describe if or how this could be done.

Response: Yes, the parametric analysis presented in Section 4 included integral results for the more superficial layer, layer I, that were shortly discussed. The additional supplementary figures in the revised version put slightly more emphasis on the modelling of layer I.

Changes made: In response to this and the previous comments, Fig S4 has been added and the corresponding discussion has been slightly enriched (see lines 617-649).

Comment 6: Define variable s and vector n for completeness, especially since n is also used for other purposes. Probably also Card (cardinality).

s was defined before first occurrence in Eq. 3 (see line 156 of the revised version). Explicit definition of vector n, besides schematics in Fig. 1a, has been added (see line 142 and revised legend of Fig. 1). Explicit definition of Card has also been added (see line 147).

Comment 7: In in several instances like Eq 24 is likely natural log and should not be italicized.

This has been corrected.
